# Why Spermatic Cord Lipomas Must be Treated as “True” Inguinal Hernias

**DOI:** 10.7759/cureus.15781

**Published:** 2021-06-20

**Authors:** Christophe R Berney

**Affiliations:** 1 Surgery, Bankstown Hospital, University of New South Wales, Sydney, AUS

**Keywords:** cord lipoma, indirect inguinal hernia, tep, tapp, recurrence, pseudo-recurrence, groin pain, endoscopic, laparoscopic

## Abstract

Lipomas of the cord are common and generally associated with an indirect hernia sac, but not always, as the lipoma may be the only pathology identified during groin exploration. Missed lipoma of the spermatic cord is unfortunately not infrequent and may lead to persistence of post-operative groin pain, with confirmation of unresected cord lipoma on postoperative ultrasound, often necessitating reoperation.

We present an interesting case of a 40-years-old male with symptomatic re-recurrent left inguinal hernia following previous open and subsequent endoscopic totally extraperitoneal (TEP) mesh repair. At laparoscopy, the previously inserted extraperitoneal mesh seemed well integrated with no evidence of recurrent hernia sac. Further transabdominal preperitoneal (TAPP) approach identified a moderate-size cord lipoma that had been missed twice before. His postoperative recovery was uneventful, and his previous symptoms completely subsided.

This is of significant value as lipomas of the cord may sometimes represent the only source of chronic groin pain in patients with no other clinical findings. Consequently, they should be viewed and treated as “true” inguinal hernias as per the European Hernia Society (EHS).

During every inguinal hernia case, the surgeon must perform rigorous exploration of the inguinal canal, looking for any herniated adipose tissue that can be easily cleared by either reduction or resection. This is further supported by both the European Association of Endoscopic Surgery (EAES) and the International Endohernia Society (IEHS) who recommend an active search for spermatic cord lipomas in all laparo-endoscopic inguinal hernia repairs.

## Introduction

Lipomas of the cord (in men) and round ligament (in women) represent a simple protrusion (or herniation) of pedunculated extra-peritoneal fat through the inguinal canal. They are quite common and since their blood supply originates from the preperitoneal space, can be simply reduced without the need to be excised [1]. Their presence is often discovered incidentally at the time of hernia repair and generally associated with an indirect hernia sac, but not always as the lipoma may be the only pathology identified during groin exploration.

Missed lipoma of the spermatic cord is a pitfall unique to the laparoscopic TAPP hernia repair, especially in the presence of sacless sliding fatty inguinal hernias where the extraperitoneal space is likely not to be inspected intraoperatively, as the peritoneum seems intact [2,3]. This false reassurance may lead to persistence of groin pain with confirmation of unresected cord lipoma on postoperative ultrasound [4]. Also less likely, this type of mishap may also occur during an endoscopic TEP approach if simply overlooked by the operating surgeon [3,4]. In both situations, missed or inadequately treated spermatic cord lipomas may suggest early recurrence or pseudo-recurrence necessitating reoperation [1].

## Case presentation

**Figure 1:**
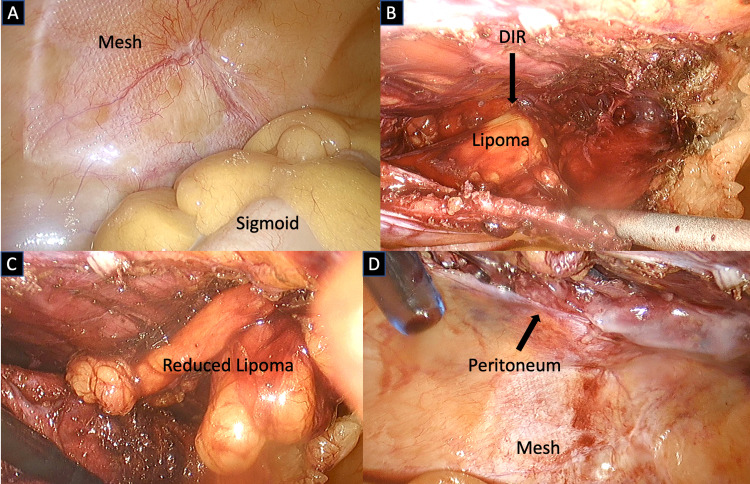
Laparoscopic revision left groin post TEP and open inguinal hernia repair A) Intact mesh in the left extraperitoneal space behind sigmoid colon; B) TAPP approach showing "overlooked" lipoma of the cord in deep inguinal ring (DIR); C) Reduced large cord lipoma; D) Laparoscopic view left groin after transverse closure peritoneum

A 40-years-old male patient was referred to our Hernia Unit with a symptomatic recurrent left inguinal hernia following a previous open left inguinal herniorrhaphy 15 years prior and more recently, endoscopic totally extraperitoneal (TEP) mesh repair of bilateral (recurrent left) inguinal hernias. At laparoscopy, the previously inserted mesh in his left groin seemed well integrated with no evidence of recurrent hernia sac (Figure 1A). Nevertheless, this was followed by a transabdominal preperitoneal (TAPP) approach that demonstrated the presence of a moderate-size cord lipoma that was easily reduced (Figure 1B, 1C). A plug was inserted in the deep inguinal ring and fixed with fibrin glue. The transverse peritoneum incision was also closed with fibrin glue (Figure 1D). His postoperative recovery was uneventful, and his previous symptoms completely subsided. During both of his previous hernia repairs, this relatively common pathology was simply overlooked, thus leading to a third totally preventable operation.

## Discussion

In a retrospective review of 280 hernia repairs, Lilly et al found 63 lipomas of the cord that represents an incidence of 22.5% [5]. Similar results were obtained by others with an incidence of 21% [6]. It is likely that the frequency may significantly increase depending on how actively we are looking for it, as shown by Carilli et al who reported an incidence of cord lipoma associated with indirect inguinal hernia of 72.5% [7]. Interestingly, when looking at the prevalence of single spermatic cord lipoma, meaning without an indirect hernia sac, both studies reported an incidence of 6.4% and 8% respectively [5,6]. Others reported slightly lower frequency ranging between 1-2.9% [8-9].

This is of significant value as lipomas of the cord or round ligament can mimic the diagnosis of inguinal hernia and may sometimes represent the only source of chronic groin pain in patients with no other clinical findings, especially in women [4,5]. Consequently, they should be viewed and treated as “true” inguinal hernias [4]. Furthermore, knowing that the prevalence of pure “sacless” spermatic cord lipoma may range between 1-8%, this diagnosis should always be clinically suspected in the presence of typical groin symptoms, especially if there is no palpable lump. In this situation, pre-operative ultrasound examination is recommended. In fact, during every inguinal hernia case, the surgeon must think about cord lipoma and perform rigorous exploration at the deep internal ring and inguinal canal, looking for any herniated adipose tissue that can be cleared by either reduction or resection [7].

Finally, we should be reminded that in the absence of a real hernia sac, a spermatic cord lipoma is still classified as a lateral (or indirect) inguinal hernia with a defect size <1.5 cm (L1) as per the European Hernia Society (EHS) [10]. Furthermore, both the European Association of Endoscopic Surgery (EAES) and the International Endohernia Society (IEHS) recommend an active search for spermatic cord lipomas in all laparo-endoscopic inguinal hernia repairs [11,12].

## Conclusions

With the rapid adoption of laparoscopic TAPP and endoscopic TEP techniques worldwide for inguinal hernia repairs, the risk of overlooking this benign pathology is noteworthy and could potentially lead to unnecessary reoperation.

From an educational point of view, it is important to readdress this matter and remind our readers that lipoma of the cord is currently classified as an indirect inguinal hernia, irrespective of the presence of a sac, and should be actively searched during every laparo-endoscopic inguinal hernia repair, as per the EAES and IEHS recommendations.
